# Supplementary material for: Current Opinion in LAIV: A Matter of Parent Virus Choice
Source: Int J Mol Sci. 2022 Jun 19;23(12):6815. doi: 10.3390/ijms23126815 (PMC9224562; doi:10.3390/ijms23126815)
Supplement: Supplementary file 1 [file ijms-23-06815-s001.zip › ijms-1707957-supplementary.pdf]

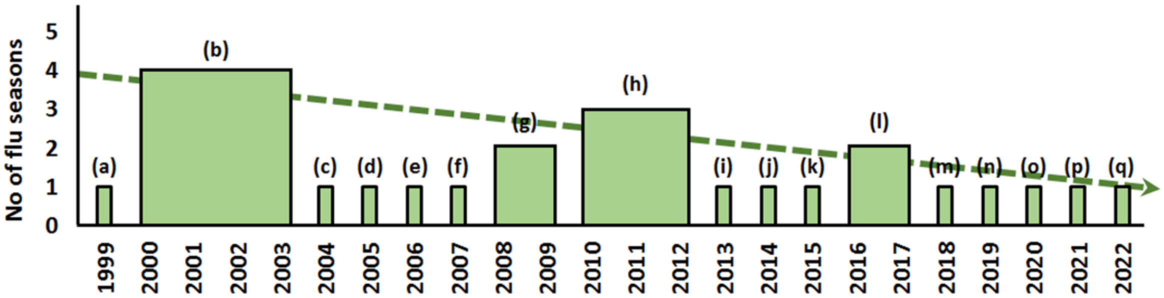

Figure S1. Recommended influenza A(H3N2) virus vaccines for use in Southern Hemisphere influenza seasons (based on [26]). A(H3N2) strains: (a) – A/Sydney/5/97 (H3N2)–like virus; (b) – A/Moscow/10/99 (H3N2)–like virus; (c) – A/Fujian/411/2002 (H3N2) – like virus; (d) – A/Wellington/1/2004 (H3N2)–like virus; (e) – A/California/7/2004 (H3N2)–like virus; (f) – A/Wisconsin/67/2005 (H3N2)–like virus; (g) – A/Brisbane/10/2007 (H3N2)–like virus; (h) – A/Perth/16/2009 (H3N2)–like virus; (i) – A/Victoria/361/2011 (H3N2)–like virus; (j) – A/Texas/50/2012 (H3N2)–like virus; (k) – A/Switzerland/9715293/2013 (H3N2)–like virus; (l) – A/Hong Kong/4801/2014 (H3N2)–like virus; (m) – A/Singapore/INFIMH–16–0019/2016 (H3N2)–like virus; (n) – A/Switzerland/8060/2017 (H3N2)–like virus; (o) – A/South Australia/34/2019 (H3N2)–like virus; (p) – A/Hong Kong/2671/2019 (H3N2)–like virus; (q) – A/Darwin/9/2021 (H3N2)-like virus. Axis X: Southern Hemisphere influenza seasons.

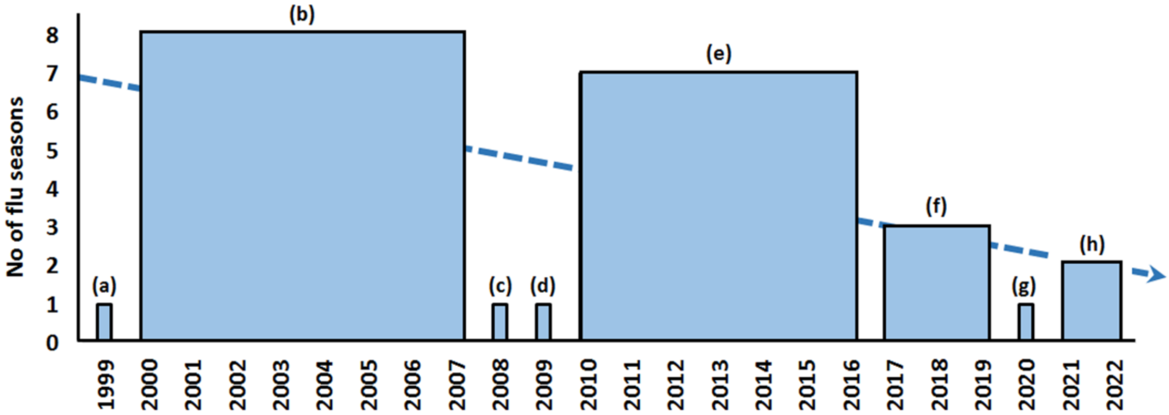

Figure S2. Recommended influenza A(H1N1) virus vaccines for use in Southern Hemisphere influenza seasons (based on [26]). A(H1N1) strains: (a) – A/Beijing/262/95 (H1N1)–like virus; (b) – A/New Caledonia/20/99 (H1N1)–like virus; (c) – A/Solomon Islands/3/2006 (H1N1)–like virus; (d) – A/Brisbane/59/2007 (H1N1)–like virus; (e) – A/California/7/2009 (H1N1)pdm09–like virus; (f) – A/Michigan/45/2015 (H1N1)pdm09–like virus; (g) – A/Brisbane/02/2018 (H1N1)pdm09–like virus; (h) – A/Victoria/2570/2019 (H1N1)pdm09–like virus. Axis X: Southern Hemisphere influenza seasons.

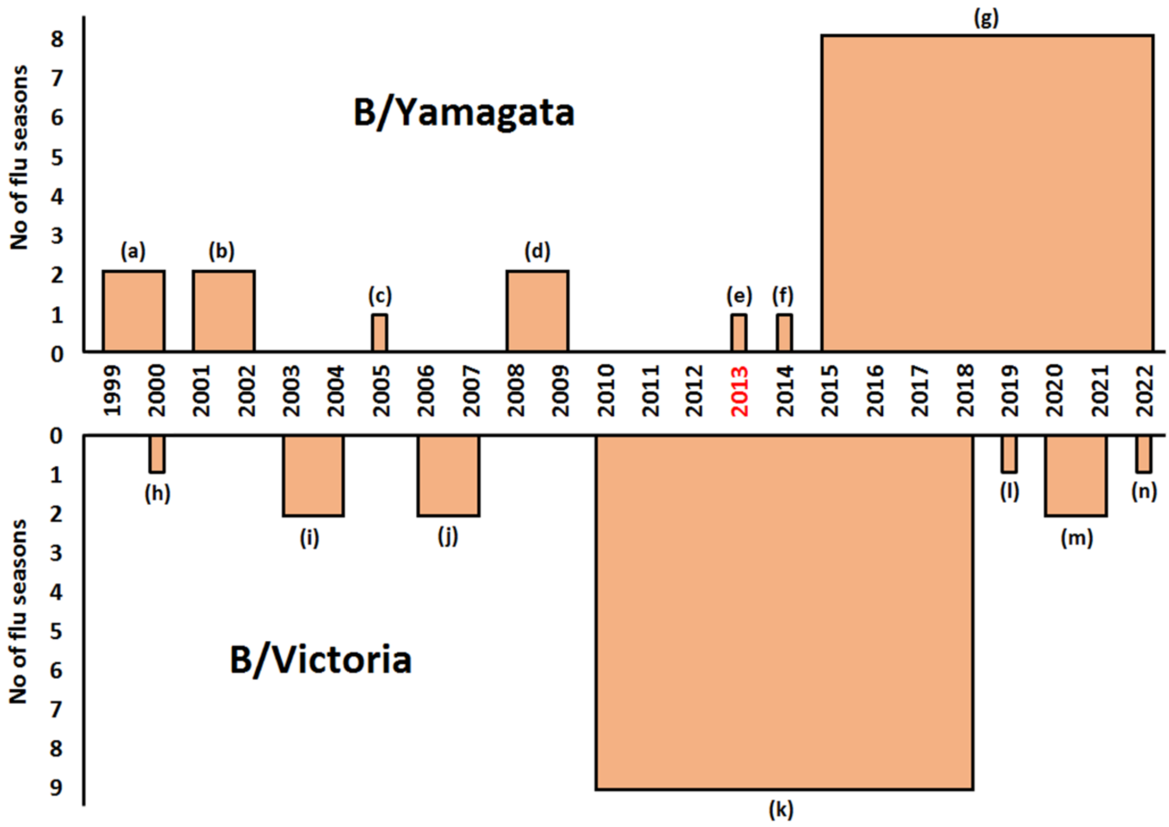

Figure S3. Recommended influenza B virus vaccines for use in Southern Hemisphere influenza seasons (based on [26]). B/Yamagata strains: (a) – B/Beijing/184/93–like virus; (b) – B/Sichuan/379/99–like virus; (c) – B/Shanghai/361/2002–like virus; (d) – B/Florida/4/2006–like virus; (e) – B/Wisconsin/1/2010–like virus; (f) – B/Massachusetts/2/2012–like virus; (g) – B/Phuket/3073/2013–like virus. B/Victoria vaccine strains: (h) – B/Shangdong/7/97-like virus; (i) – B/Hong Kong/330/2001–like virus; (j) – B/Malaysia/2506/2004–like virus; (k) – B/Brisbane/60/2008–like virus; (l) – B/Colorado/06/2017–like virus; (m) – B/Washington/02/2019–like virus; (n) – B/Austria/1359417/2021-like virus. 2013 influenza season (highlighted in red) when the first WHO recommendations for the composition of a quadrivalent influenza vaccine were made [26]. Axis X – Southern Hemisphere influenza seasons.
